# Fusion-Negative NTRK Overexpression Exhibit Biological Relevance in Colorectal Cancer: Implications for Prediction of Responses to Kinase Inhibitors

**DOI:** 10.3390/ph18101562

**Published:** 2025-10-16

**Authors:** Abdulaziz Alfahed

**Affiliations:** Department of Medical Laboratory, College of Applied Medical Sciences, Prince Sattam bin Abdulaziz University, Al-Kharj 11942, Saudi Arabia; a.alfahed@psau.edu.sa

**Keywords:** colorectal cancer, fusion-negative NTRK signalling, *NTRK1*, *NTRK2*, *NTRK3*, cryptic *NTRK3* fusion, cancer pathways cross-talks, kinase inhibitor response, clinicopathological and molecular features

## Abstract

**Background/Objectives**: The aims of this study are to define the roles of the neurotrophic tyrosine receptor kinase genes *NTRK1*, *NTRK2* and *NTRK3* (*NTRK1/2/3*) in CRC and to determine the clinicopathological, molecular, cancer signalling and potential predictive significances of *NTRK1/2/3* expression in CRC, irrespective of NTRK gene fusion. **Methods**: Standard statistical tests in SPSS were utilised to interrogate the associations and correlations between *NTRK1/2/3* expression and clinicopathological, molecular and genomic features in two CRC cohorts. *NTRK1/2/3* expression deregulation was also investigated using correlation and regression analyses. Furthermore, gene set enrichment analysis (GSEA) and pathway/drug ontology enrichment analysis (POEA/DOEA) were utilised to interrogate the enrichment of cancer signalling pathways, as well as NTRK and other tyrosine kinase inhibitor response in the CRC cohorts. **Results**: Whilst *NTRK1* expression was higher in the CRC subset with microsatellite instability, *NTRK2/3* expression was preferentially overexpressed in the microsatellite stable subsets. Moreover, there was differential *NTRK1/2/3* expression with respect to clinicopathological and molecular/genomic indices. In addition, this study demonstrated that *NTRK1/2/3* expression was deregulated by a combination of copy number alterations (*NTRK2*), aberrant methylation (*NTRK1/2/3*) and potentially and cryptic gene fusion (*NTRK3*). Furthermore, GSEA and POEA demonstrated that *NTRK1/2/3*-high CRC subsets exhibited enrichment of and cross-talks among the NTRK signalling pathways, as well as of known cancer signalling pathways. The GSEA and DOEA showed that NTRK signalling was enriched for kinase inhibitors responses, representing evidence that *NTRK1/2/3* expression may serve as biomarkers for multiple kinase inhibitors, including entrectinib—the tissue-agnostic kinase inhibitor for cancers with NTRK gene fusions. **Conclusions**: The results demonstrated that fusion-negative NTRK signalling may be active in CRC and may contribute to the molecular pathogenesis and biology of the disease. The results also demonstrated that the *NTRK1/2/3* expression may be predictive multiple kinase inhibitors.

## 1. Introduction

Colorectal cancer (CRC) remains a disease of public health concern worldwide, inasmuch as its epidemiological profile remains dismal. CRC represents the fourth most common cancer and the second most common cause of cancer deaths [1]. Despite the large amount of capital resources expended to understand the epidemiology and molecular pathogenesis of the disease [2]), the incidence and mortality rates have remained high [1,3,4]. Hence, continuous research aimed at understanding the drug susceptibility of the disease should be encouraged.

This study examines the expression of neurotrophic tyrosine receptor kinase (NTRK) genes *NTRK1*, *NTRK2* and *NTRK3* (*NTRK1/2/3*), in CRC cohorts, with respect to their potential biological roles in the disease. Physiologically, *NTRK1/2/3*, which encode tyrosine receptor kinases TrkA (*NTRK1*), TrkB (*NTRK2*) and TrkC (*NTRK3*), function in the neurotrophin signalling pathways, wherein they mediate differential biological functions [5,6]. For example, TrkA, which is activated by nerve growth factors (NGFs) and neurotrophin-3 (NT-3), mediates nerve axon growth, cell survival and suppression of neuron apoptosis following insults from axotomy, ischaemia and oxidative stress [5,7]. These actions are mediated through recognized cancer signalling pathways such as PI3K, Akt, Ras and MAPK signalling pathways [5,7]. TrkB, on the other hand, is activated by brain-derived neurotrophic factor (BDNF)- and neurotrophin-4 (NT-4)-binding and has been demonstrated in epilepsy studies to function in neural kindling [5,7]. The functions of TrkC are mediated by proteins containing phosphotyrosine and SH2 domains following TrkC binding by NT-3 [5].

The clinical importance of NTRK genes in cancer lies in the gene fusions that they form with multiple 5′ partner genes [5,7,8]. These gene fusions result in the overexpression of the fusion proteins, which incorporate the ligand-independent, constitutively active TrkA, TrkB and TrkC proteins that drive cancer growth [5,7,8]. Furthermore, these receptor kinases are the targets of the NTRK inhibitors (NTRKi’s), such as entrectinib, larotectinib and zarlutrectinib [5,7,8]. While NTRK fusions have been well established as oncogenic drivers through constitutive activation of TRK signalling [5,7,8], recent evidence indicates that NTRK overexpression can also occur independently of gene fusion events. Such fusion-negative NTRK overexpression represents a distinct and less-explored mechanism of dysregulation, potentially arising from alternative genomic or epigenetic alterations such as gene amplification or promoter hypomethylation [9]. Fusion-independent TRK expression has been reported in several malignancies, including non-small-cell lung cancer, dedifferentiated liposarcoma, and pancreatic neuroendocrine tumours, where overexpression was observed in the absence of detectable gene fusions [6,10,11]. However, current evidence regarding the therapeutic significance of fusion-independent NTRK overexpression is very limited. While the proven efficacy of TRK inhibitors such as larotrectinib and entrectinib in fusion-positive tumours demonstrates that TRK signalling is a therapeutically targetable pathway, no clinical studies have yet confirmed similar responses in fusion-negative settings.

The aim of this study is to explore the biological roles of fusion-negative *NTRK1/2/3* overexpression in CRC, with a view to determining their potential predictive role for kinase inhibitor (including NTRKi) responses in CRC. The specific study objectives include determining (i) the clinicopathological features of NTRK gene expression in CRC, (ii) the molecular/genomic correlates of NTRK gene expression in CRC and (iii) the mechanisms of *NTRK1/2/3* expression deregulation in CRC; assessing (iv) the enrichment and activation of *NTRK1/2/3* signalling in CRC; and investigating (v) the potential for *NTRK1/2/3* expression to predict kinase inhibitor response in CRC. This study’s hypothesis is that *NTRK1/2/3* expression, outside the context of gene fusion, may play biological roles in CRC, which may include influencing the clinicopathological presentation, regulation of molecular and signalling pathways and functioning as potential predictor of kinase inhibitor response in CRC.

## 2. Results

There was a significant correlation among *NTRK1/2/3* expression (*NTRK1* vs. *NTRK2*: Spearman’s rho = 0.105, *p* = 0.002; *NTRK1* vs. *NTRK3*: Spearman’s rho = 0.289, *p* < 0.001; *NTRK2* vs. *NTRK3*: Spearman’s rho = 0.236, *p* < 0.001). *NTRK1/2/3* expression was dichotomised using the median cut-off of the combined NTRK gene expression data.

### 2.1. Clinicopathological and Molecular Correlates of NTRK1/2/3 Expression in CRC

The clinicopathological and molecular correlates of *NTRK1/2/3* expression were interrogated in the combined TCGA and Sidra_LUMC CRC cohort using a chi-square test, followed by binary logistic regression, to exclud cohort bias. The results demonstrated differential correlations between individual NTRK gene expression levels and specific clinical and molecular features of CRC.

*NTRK1* expression exhibited a significant association with age at diagnosis, tumour location, pathological tumour (T) stage, pathological metastasis (M) stage, microsatellite instability status, molecular subtype according to the epithelial (CIN)/hypermutated (MSI)/mesenchymal (genome-stable, GS) scheme, *BRAF* mutation status, fraction genome altered (FGA) state and tumour mutational burden (TMB). However, there was no association between *NTRK1* expression and gender, histological type, pathological nodal stage (N), *TP53* mutation status, and overall TNM stage. Moreover, there was no association between *NTRK1* expression and overall survival (OS) or and disease-free survival (DFS) (OS log-rank test: X^2^ = 0.001, *p* = 0.973; DFS log-rank test: X^2^ = 0.595, *p* = 0.441). Specifically, *NTRK1* expression was significantly higher in right-sided tumours, late pathological T stage, non-metastasis (M0) stage, MSI positive state, the hypermutated/MSI subtype, *BRAF* mutation-positive, low FGA state, low aneuploidy state and high TMB. Furthermore, a higher fraction of *NTRK1*-high cases was found in the mucinous type of CRC, but this association did not achieve statistical significance (Supplementary Materials 1).

*NTRK2*, in contrast, demonstrated higher expression in lower nodal (N0) stage, MSS state, the mesenchymal/genome-stable subtype (compared to hypermutated/MSI) and low aneuploidy states, but not in the other features examined (Supplementary Materials 2). Furthermore, high *NTRK2* expression showed association with shorter overall and disease-free survival (OS log-rank test: X^2^ = 6.126, *p* = 0.013; DFS log-rank test: X^2^ = 11.234, *p* < 0.001) in univariate analysis (irrespective of CRC cohort), but these associations were not independent of tumour, nodal or metastatic disease stages.

On the other hand, *NTRK3* expression displayed higher expression in left-sided tumours, higher nodal stage (≥N1), late overall (TNM) disease stage, MSS state and low TMB cases. *NTRK3* expression showed a significant association with *TP53* mutation status in univariate analysis, but this association did not achieve statistical significance on correction for multiple testing. There was no significant difference in *NTRK3* expression between the mesenchymal/GS and epithelial/CIN subtypes of CRC. Furthermore, there was no association between *NTRK3* expression and the other clinicopathological features (Supplementary Materials 3), or with survival profiles (OS log-rank test: X^2^ = 0.103, *p* = 0.748; DFS log-rank test: X^2^ = 3.498, *p* = 0.061).

Overall, *NTRK1/2/3* exhibited molecular subtype-specific expression and some differential clinicopathological features, evidence that the NTRK genes may have differential biological roles in CRC.

### 2.2. NTRK1/2/3 Expression Deregulation in CRC

#### 2.2.1. *NTRK1/2/3* Copy Number Alterations

*NTRK1/2/3* copy numbers were retrieved from the gene copy number datasets for the TCGA and Sidra-LUMC CRC cohorts. For *NTRK1*, there were 69/805 (8.6%), 552/805 (68.6%) and 184/805 (22.9%) cases with loss/deletion, wild-type and gain/amplification, respectively. *NTRK2* had 120/805 (14.92%), 557/805 (69.2%) and 128/805 (15.9%) cases with loss/deletion, wild-type and gain/amplification, respectively, while *NTRK3* had 253/805 (31.4%), 465/805 (57.8%) and 90/805 (11.2%) cases with loss/deletion, wild-type and gain/amplification statuses.

**Figure 1:**
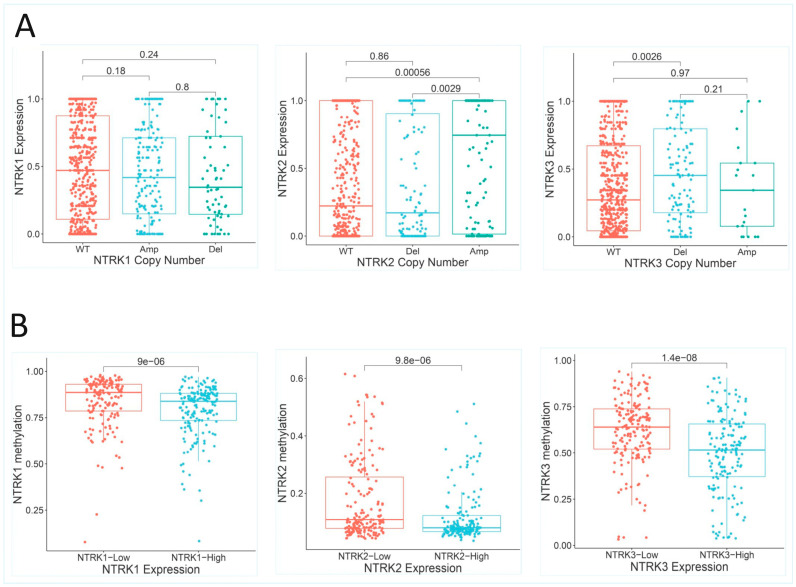
Box plots demonstrating the *NTRK1/2/3* expression deregulation in the TCGA and Sidra-LUMC cohorts by copy number changes and abnormal methylation (**A**). Deregulation of *NTRK1/2/3* expression by copy number changes demonstrating that whilst *NTRK2* expression is altered by copy number in a canonical pattern, *NTRK3* expression alteration by copy number occured in a paradoxical pattern: *NTRK3* deletion was associated with high *NTRK3* expression (**B**). Deregulation of *NTRK1/2/3* expression by altered methylation in the TCGA cohort followed a canonical pattern. Copy number alterations status: Del = deletions, Amp = amplifications and WT (wild-type) = copy number-normal.

There was no association between *NTRK1* copy number and *NTRK1* expression (Figure 1A). However, there was direct association between *NTRK2* copy number and *NTRK2* expression (wild-type vs. gain/amplification: *p* < 0.001; loss/deletion vs. gain/amplification: *p* = 0.003; loss/deletion vs. wildtype: *p* = 0.860) (Figure 1A). On the other hand, *NTRK3* copy number demonstrated a paradoxical relationship with *NTRK3* expression: there was increased proportion of cases with *NTRK3* loss/deletion relative to wild-type cases in the *NTRK3* expression-high subset (*p* = 0.003) (Figure 1A); however, there was no difference in *NTRK3* expression between *NTRK3* loss/deletion and *NTRK3* gain/amplification (*p* = 0.210) or between *NTRK3* gain/amplification and the *NTRK3* wild-type (*p* = 0.970) cases. The overall results demonstrated that copy number alteration may be more important in the deregulation of *NTRK2* and *NTRK3* expression than in the deregulation of *NTRK1* expression.

#### 2.2.2. Potential *NTRK3* Gene Fusion

**Figure 2:**
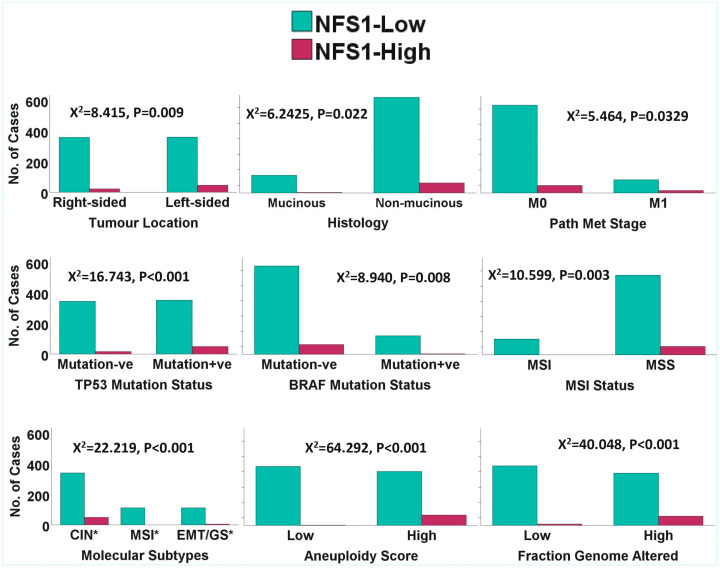
Clustered bar charts demonstrating associations of the *NTRK3* fusion score 1 (NFS1) with clinicopathological and molecular indices of CRC. Only significant associations are shown here. The stated *p* values are false discovery rates. The significant associations of NFS1 with clinicopathological features validated the biological relevance of NFS1 in CRC pathogenesis. CIN* = epithelial/chromosomal instability subtypes; MSI* = hypermutated/MSI; EMT/GS* = mesenchymal/EMT/genome-stable subtypes.

**Figure 3:**
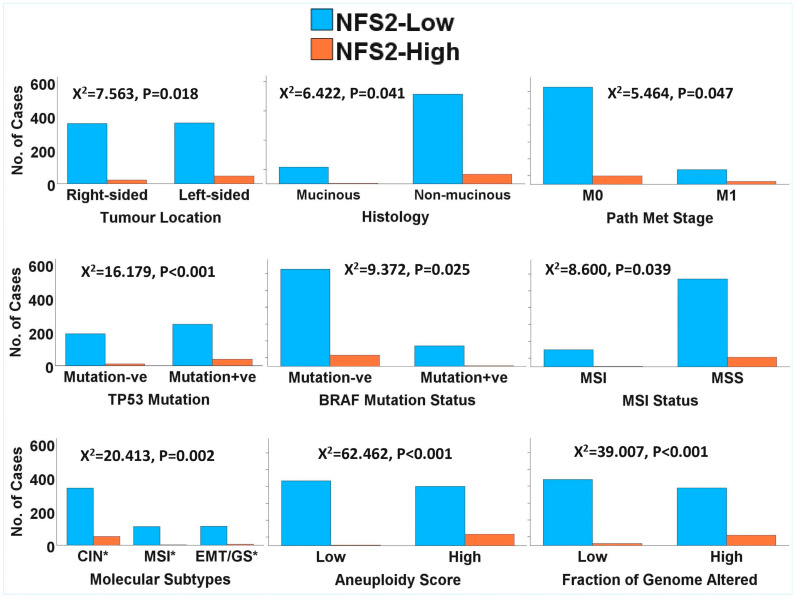
Clustered bar charts showing the relationships between *NTRK3* fusion score 2 (NFS2) and clinicopathological and molecular indices of the TCGA and Sidra-LUMC cohorts of CRC. The associations between NFS2 and clinical, pathological and molecular features of NFS1 suggest that NFS2 may denote *NTRK3* cryptic gene fusion. Only significant associations are shown here. The stated *p* values are false discovery rates. CIN* = epithelial/chromosomal instability subtypes; MSI* = hypermutated/MSI; EMT/GS* = mesenchymal/EMT/genome-stable subtypes.

The paradoxical relationship between *NTRK3* copy number and gene expression was further investigated using the TCGA and Sidra-LUMC copy number segment datasets and a more stringent segment mean threshold of −0.3 for gene deletion. The *NTRK3* gene was interrogated for potential participation in gene fusion based on the notion that partial deletion of the 5′ portion of the 3′ fusion partner is associated with overexpression of that 3′ partner, especially in the presence of 3′ deletion of a 5′ partner. *NTRK3* was probed for any deletion associated with deletions of the common 5′ fusion partners of *NTRK3*, namely *ETV6* (*TEL*), *TPM3*, *TPR*, *SQSTM1*, *EML4*, *MYH9* and *MYO5A*. *NTRK3* fusion was inferred when an individual case exhibited high *NTRK3* expression in association with *NTRK3* and known 5′ partner deletion (*NTRK3* fusion score (NFS) 1), or when there was an association between high *NTRK3* expression and *NTRK3* deletion alone (NFS2). The results demonstrated that 118/805 cases exhibited deletions of *NTRK3* and one or the other 5′ partner of the *NTRK3* fusion. In addition, 296/805 cases exhibited deletions of *NTRK3* or one of the 5′ partner genes, while 120/805 cases had *NTRK3* deletions alone. Multiple linear regression analysis with control for cohort bias showed that CRC cases with *NTRK3* deletions exhibited higher *NTRK3* expression than the cases without *NTRK3* deletions (*p* = 0.002), as did the double *NTRK3*-fusion partner deletions (*p* = 0.003). However, an analysis of the cases with the 5′ partners deletion alone did not show any significant difference in *NTRK3* expression compared to cases without 5′ partner deletions (*p* = 0.143). Furthermore, 68/805 (8.44%) and 70/805 (8.70%) cases exhibited high NFS1 (NFS1 = 3) (*NTRK3* deletion + any 5′ partner deletion + high *NTRK3* expression) and high NFS2 (NFS2 = 2) (*NTRK3* deletion + high *NTRK3* expression) scores. This rate is significantly higher than the <1% frequency found for the entire *NTRK1/2/3* fusions in CRC. NFS1 and NFS2 were also interrogated for any relationship with clinicopathological and molecular features of CRC. The results demonstrated that both fusion scores exhibited significant relationships with clinical, pathological and molecular parameters of CRC tested, thereby validating them as relevant biological factors in CRC pathogenesis (see Figure 2 and Figure 3). The findings also suggested that NFS1 and NFS2 may denote *NTRK3* cryptic gene fusion. Interestingly, NFS1 and NFS2 exhibited significant associations with a greater number of clinicopathological and molecular features than *NTRK3* expression (Supplementary Materials 4 and Supplementary Materials 5).

#### 2.2.3. *NTRK1/2/3* Methylation Status

The TCGA methylation data were interrogated for the methylation status of *NTRK1/2/3* and for the contribution of abnormal methylation to *NTRK1/2/3* expression deregulation. No methylation data was available for the Sidra-LUMC cohort. There was a canonical (indirect) correlation between gene expression and methylation status (NTRK1: Spearman’s rho = −0.286, *p* < 0.001; *NTRK2*: Spearman’s rho = −0.284, *p* < 0.001; *NTRK3*: Spearman’s rho = −0.340, *p* < 0.001) (Figure 1B). Multiple linear regression analysis (while controlling for cohort bias and *NTRK1* copy number alterations: adjusted R2 = 0.051, F = 10.022, *p* < 0.001) demonstrated that *NTRK1* methylation predicted *NTRK1* expression independently (*p* < 0.001, B = −0.611). As before, *NTRK1* copy number did not significantly contribute to differences in *NTRK1* expression (*p* = 0.104, B = 0.059). Furthermore, *NTRK2* methylation exhibited significant correlations with *NTRK2* expression (adjusted R2 = 0.118, F = 23.548, *p* < 0.001), also in a canonical pattern (*p* < 0.001, B = −1.042), independent of cohort bias and *NTRK2* copy number alterations (*p* < 0.001, B = 0.177). Moreover, multiple linear regression analysis of *NTRK3* methylation (adjusted R2 = 0.336, F = 58.123, *p* < 0.001) demonstrated that *NTRK3* methylation predicted *NTRK3* expression in a canonical pattern (*p* < 0.001, B = −0.531) and independent of copy number alterations and *NTRK3* fusion scores. The *NTRK3* fusion score (*p* < 0.001, B = 0.209) and *NTRK3* copy number alterations (*p* = 0.003, B = 0.095) also exhibited independent correlations with *NTRK3* expression. In summary, this study demonstrated that abnormal methylation, but not copy number alterations, contributed to the deregulation of *NTRK1* expression. In addition, both abnormal methylation and copy number alterations independently predicted *NTRK2* expression, while methylation status, copy number alterations, and potential *NTRK3* gene fusion significantly contributed to *NTRK3* deregulation in CRC.

### 2.3. Enrichment of NTRK1/2/3 and Other Cancer Signalling Pathways in the CRC Cohorts

**Figure 4:**
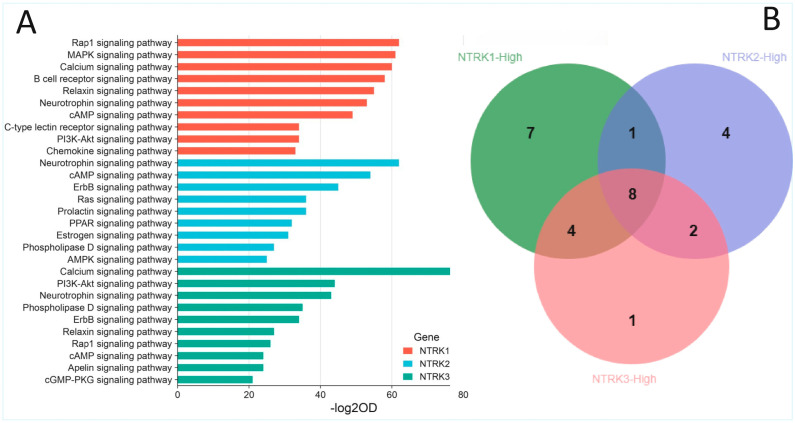
Enrichment of NTRK and cancer signalling in *NTRK1/2/3*-high subsets of CRC (**A**). Bar charts showing the enrichment of NTRK (neurotrophin) and other cancer signalling pathways in the *NTRK1*-high, *NTRK2*-high and *NTRK3*-high subsets of CRC. Only the top 10 significantly enriched pathways in each NTRK-high category are shown here (see Supplementary Materials 6–8 for details) (**B**). Venn diagram showing cancer signalling pathways which were commonly enriched in the *NTRK1*-high, *NTRK2*-high and *NTRK3*-high subsets of the CRC subsets.

**Figure 5:**
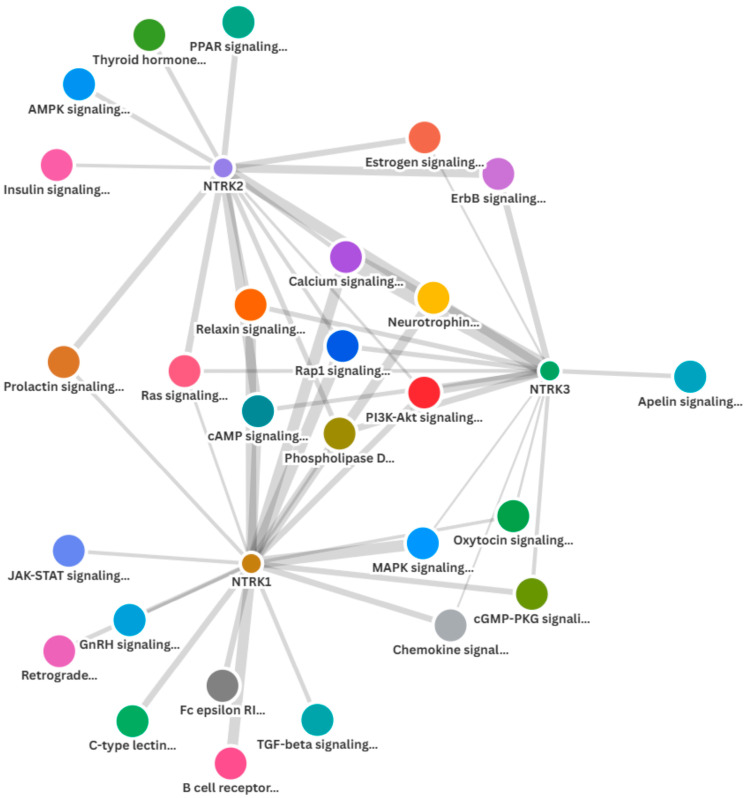
Network graph demonstrating interactions and cross-talks among the enriched cancer signalling pathways in the *NTRK*-high subsets of CRC.

The GSEA, followed by the GOEA, demonstrated that *NTRK1/2/3* signalling pathways were over-represented in the *NTRK1/2/3*-high subsets of both CRC cohorts. Furthermore, there were interactions among the three pathways, as demonstrated by the enrichments of all *NTRK1/2/3* signalling pathways (Reactome pathways 2024) in each of the *NTRK1*-, *NTRK2*- and *NTRK3*-high phenotypes but not in the low subsets. Moreover, there was enrichment of known cancer signalling pathways in the *NTRK1/2/3*-high subsets of CRC. Specifically, pathway ontology enrichment (KEGG 2021 Human) demonstrated that MAPK, PI3K-Akt, neurotrophin, JAK-STAT, Ras and TGF-beta signalling pathways, among others, were overrepresented in the *NTRK1*-high subset of both CRC cohorts but not in the *NTRK1*-low CRC subset (Figure 4A and Supplementary Materials 6). Similarly, enrichment of neurotrophin, Ras, ErbB, AMPK and PI3K-Akt signalling, among others, was found in the *NTRK2*-high subset but not in the *NTRK2*-low CRC subset (Figure 4A and Supplementary Materials 7). In addition, the *NTRK3*-high CRC subset, but not the *NTRK3*-low one, exhibited enrichment of PI3K-Akt, neurotrophin, ErbB, Ras, MAPK, chemokine and oestrogen signalling pathways, among others (Figure 4A and Supplementary Materials 8). Supplementary Materials 9 and Figure 4B showed that eight (8) cancer pathways, including the neurotrophic signalling pathways, were commonly enriched in the *NTRK1*-high, *NTRK2*-high and *NTRK3*-high subsets of the CRC cohorts. Between the *NTRK1*-high and *NTRK2*-high subsets, there was enrichment of the prolactin signalling pathway. Four (4) cancer pathways were commonly enriched between the *NTRK1*-high and *NTRK3*-high subsets, while two (2) pathways were commonly enriched between the *NTRK2*-high and *NTRK3*-high CRC subsets. The findings confirmed that *NTRK1/2/3* signalling is active in CRC and demonstrated that there is cross-talks between NTRK signalling and known cancer pathways in the CRC cohorts. Furthermore, the similarity of cancer signalling pathways upregulated by each of *NTRK1/2/3*-high subsets provides evidence of interaction among the three NTRK signalling pathways (Figure 5). Moreover, there was enrichment of all three NTRK signalling in the NFS1- and NFS2-high subsets of the CRC cohorts (all at nominal *p* values < 0.001 and FDR < 0.007) but not in the NFS1/NFS2-low subsets. Pathway ontology enrichment demonstrated upregulation of similar cancer pathways found for the *NTRK1/2/3*-high CRC subsets (Supplementary Materials 10), lending credence to the notion that NFS1 and NFS2 have valid biological roles in CRC pathogenesis and that NFS1/NFS2 may represent cryptic *NTRK3* fusion.

### 2.4. Enrichment of Responses to Multiple Tyrosine Kinase Inhibitors in the CRC Cohorts

**Figure 6:**
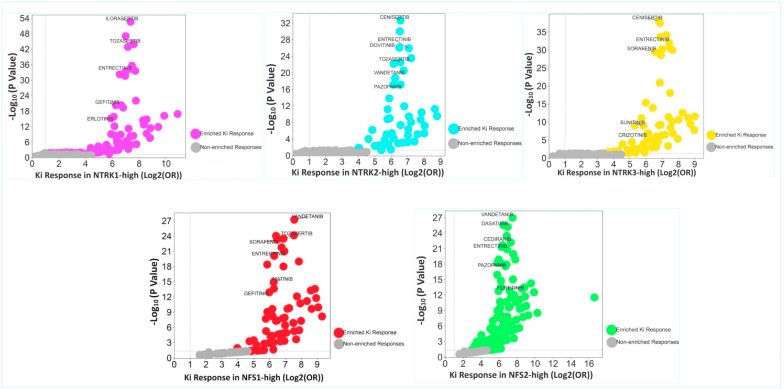
Volcano plots for *NTRK1*-high, *NTRK2*-high, *NTRK3*-high, NFS1-high and NFS2-high subsets of CRC demonstrating enrichment of responses of multiple kinase inhibitors (Ki’s), including the NTRK inhibitor. The volcano plots were generated using the log2 odds ratios (ORs) of kinase inhibitor enrichment plotted against the −log10 of the adjusted *p* values.

A total of 40 kinase inhibitor (Ki) response gene sets (https://dsigdb.tanlab.org/DSigDBv1.0/, accessed on 5 September 2025) were interrogated using the GSEA, followed by the DOEA (Enrichr/DGIdb Drug Targets 2024, accessed on 5 September 2025) for the enrichment of kinase inhibitor responses. There was enrichment of 36/40 and 0/40 Ki-response gene sets in the *NTRK1*-high and *NTRK1*-low subsets, respectively, at nominal *p* value < 5% and FDR < 25% in the TCGA cohort. The *NTRK2*-high subset of the TCGA cohort, but not the *NTRK2*-low subset, displayed enrichment of 36/40 Ki-response gene sets. In addition, enrichment of 37/40 and 0/40 Ki-response gene sets at nominal *p* value < 5% and FDR < 25% were observed in the *NTRK3*-high and *NTRK3*-low subsets, respectively, in the TCGA cohort. In the Sidra-LUMC cohort, 32/40 and 0/40 Ki-response gene sets were enriched in the *NTRK1*-high and *NTRK1*-low, respectively, at a nominal *p* value < 5% and FDR < 25%. In addition, enrichment of 35/40 and 0/40 Ki-response gene sets occurred in the *NTRK2*-high and *NTRK2*-low subsets, respectively, at nominal *p* value < 5% and FDR < 25%, while 34/40 and 0/40 gene sets, respectively, were enriched in the *NTRK3*-high and *NTRK3*-low subsets at nominal *p* value < 5% and FDR < 25%. Drug ontology enrichment analysis demonstrated enrichment of responses for multiple kinase inhibitors for each of *NTRK 1/2/3*-high subsets in the combined CRC cohorts. The top 10 kinase inhibitor ontology terms enriched in the *NTRK1*-high subset included Ilorasertib, Cenisertib, Tozasertib, Dovitinib, Entrectinib, Vandetanib, Cediranib, Dasatinib, Sorafenib and Linifanib (see Supplementary Materials 11 and Figure 6). The most common kinase inhibitor ontologies enriched in the *NTRK2*-high subset are Cenisertib, Ilorasertib, Dovitinib, Entrectinib, Cediranib, Tozasertib, Sorafenib, Vandetanib, Dasatinib and Pazopanib (see Supplementary Materials 12 and Figure 6), while the most common inhibitor terms enriched in the *NTRK3*-high subset include Cenisertib, Ilorasertib, Tozasertib, Dovitinib, Entrectinib, Vandetanib, Dasatinib, Cediranib, Sorafenib and Linifanib (see Supplementary Materials 13 and Figure 6). Overall, the results added credence to my position that an interplay exists among the NTRK pathways. They also confirmed the interplay between the NTRK pathways and other kinase signalling pathways, but more importantly, the results showed that *NTRK1/2/3* expression may serve as a predictive biomarker for response to multiple kinase inhibitors.

### 2.5. Enrichment of Kinase Inhibitor Responses in NFS1/2-High Subsets

Enrichment of kinase inhibitor response was interrogated by the GSEA and DOEA in the CRC cohorts by using the NFS1/2 scores as phenotypes. The results showed enrichment of 21/40 and 0/40 Ki-response gene sets in both NFS1/NFS2-high and NFS1/NFS2-low subsets, respectively, in the Sidra-LUMC CRC cohort. In the TCGA cohort, enrichment of 29/40 and 1/40 Ki-response gene sets was observed in the NFS1-high and NSF1-low subsets, respectively. In addition, enrichment of 26/40 and 0/40 gene sets was found in the NFS2-high and NFS2-low subsets, respectively. The DOEA (Enrichr/DGIdb Drug Targets 2024) of the combined CRC cohorts confirmed the enrichment of multiple tyrosine kinase inhibitors in the NFS1/2-high subsets of CRC. The top 10 enriched Ki responses observed for the NFS1-high subset included Vandetanib, Cediranib, Ilorasertib, Tozasertib, Dasatinib, Sorafenib, Linifanib, Entrectinib, Dovitinib and Regorafenib (Supplementary Materials 14 and Figure 6), while the top 10 Ki-response gene sets demonstrated for the NFS2-high subset were similar to those seen for the NFS1-high subset and included Vandetanib, Ilorasertib, Tozasertib, Dasatinib, Linifanib, Cediranib, Dovitinib, Sorafenib, Entrectinib and Regorafenib (Supplementary Materials 15 and Figure 6). The enriched Ki-response sets were also similar to those observed for *NTRK1/2/3*-high subset of the CRC cohorts.

## 3. Discussion

This study has shown that outside the context of NTRK fusion, *NTRK1/2/3* expression has biological relevance in CRC. Fusion-negative *NTRK1/2/3* expression demonstrated correlations with clinicopathological and molecular features of CRC, as well as associations with cancer-relevant signalling pathways. *NTRK1/2/3* expression demonstrated molecular subtype-specific expression: whereas high *NTRK1* expression was associated with the MSI and hypermutated subtypes of CRC, NTRK2/3 expression showed associations with the MSS subtype. *NTRK1/2/3* expression also demonstrated associations with some adverse clinicopathological features of CRC. These relationships are in keeping with the established oncogenic functions of *NTRK1/2/3* in cancers generally [5,7,8]. This is the first time any study has attempted to interrogate the biological relevancies of fusion-negative *NTRK1/2/3* expression in CRC. This study also showed that high *NTRK1/2/3* expression was associated with enrichment of known cancer signalling pathways. The relationship between *NTRK1/2/3* expression and cancer signalling pathways is fairly established. For example, it has previously been demonstrated that fusion-based overexpression of *NTRK1/2/3* drives downstream signalling through PI3K-Akt-mTOR, RAS-RAF-MEK-ERK, PLCγ-DAG-IP3 and PLCγ-PKC-Ca^2+^ pathways [5,6,7,8,9]. The findings of this study, specifically, the enrichment of PI3K-Akt, EGFR, MAPK, AMPK and ERK signalling in the *NTRK1/2/3*-high CRC subsets, are in consonance with the established signalling relationships of *NTRK1/2/3* expression with cancer signalling pathways.

Furthermore, this study showed that *NTRK1/2/3* expression was mainly deregulated by copy number and methylation mechanisms in CRC. The findings demonstrated that the main mechanisms of *NTRK1/2/3* expression deregulation in CRC are the established and most common mechanisms of gene deregulation in cancers [9]. In addition, this study showed that in about 8% of CRC cases, *NRTK3* expression may be deregulated by cryptic *NTRK3* fusion. The association between high *NTRK3* expression and *NTRK3* deletion was coded as fusion scores, NFS1 and NFS2. There was a demonstrated association between high *NTRK3* expression and *NTRK3*, as well as known *NTRK3* 5′ partner deletions. It has long been established that an association between high expression of a gene with demonstrated deletions indicated that a fusion event was associated with that gene [12,13,14,15]. This association has been used to infer the presence of gene fusions in many research studies [12,13,14,15], and in the absence of structural variations data or in the absence of demonstrable gene fusion in structural variation data, cryptic gene fusion can be inferred from the association between gene overexpression and gene deletion. The implication of the findings is that the frequency of NTRK fusion in CRC may be higher than the rates currently stated in the literature [16].

The demonstration of enrichment of response to NTRKi and other kinase inhibitors in the CRC subsets with high *NTRK1/2/3* expression has certain implications for the potential predictive values of *NTRK1/2/3* expression. The first implication is that the level of NTRK gene expression, be it in the context of gene fusion, gene amplification or aberrant methylation, may be a more relevant predictor of response to NTRK inhibition than the actual mechanisms of altered expression. This position is clearly supported by recommendations in the guidelines for biomarker testing for NTRK inhibition purposes, wherein immunohistochemistry, a protein expression technique, can be used for screening NTRK fusion in cancer types with low NTRK fusion rates and as a surrogate for NTRK fusion-detection techniques in settings where next-generation sequencing is not feasible [7,8,17]. *NTRK1/2/3* gain/amplification and aberrant methylation can increase the expression of the *NTRK1/2/3* products, as do NTRK fusions, albeit in the context of chimeric proteins [5,7,8]. Furthermore, the kinase domains present in the TrkA, TrkB and TrkC molecules are the precise targets of the NTRK inhibitors, irrespective of the mechanisms of their overexpression [5,7]; hence, *NTRK1/2/3* expression, even in the absence of NTRK fusion, may be a veritable predictor of response to these kinase inhibitors. However, to the best of my knowledge, *NTRK1/2/3* expression has yet to be tested in any clinical trial as a predictive biomarker of NTRK or other inhibitors. Secondly, the enrichment of response to multiple kinase inhibitors in the *NTRK1/2/3*-high subsets of CRC may indicate that *NTRK1/2/3* overexpression can be a predictive biomarker for these kinase inhibitors. Although further investigations, including comprehensive clinical trials, are required for their confirmations as such, it is imperative that the implications of this study’s findings be discussed here. The concept of integrated, multi-targeted approach to cancer therapy, whereby high order (≥5) combinations of targeted therapeutic agents are utilized to simultaneously target multiple biological pathways in cancer, is an enticing one [18,19]. The purpose of such strategy would be to improve the efficacy of treatment and the chances of a cure [18,19], whilst reducing the risks of therapy resistance and of drug toxicity [18,19]. However, such strategy would require a super biomarker, the expression of which denotes responses to multiple drugs. This study’s finding of enrichment of responses to multiple kinase inhibitors in *NTRK1/2/3*-high cases may indicate that the integrated therapy concept, supported by a super-biomarker framework, is feasible.

This study may be limited by the absence of structural data for the Sidra-LUMC cohort and hence the inability to exclude potential cases with NTRK fusion from that cohort. However, this study anticipated that the confounding effects of including potential NTRK fusion-positive cases (<1% of cases) were likely insignificant to the overall study analyses.

## 4. Materials and Methods

### 4.1. Study Approach

First, the clinicopathological correlates of *NTRK1/2/3* expression were interrogated using the appropriate statistical tests in SPSS version 29. Then, the mechanisms of *NTRK1/2/3* expression deregulation were interrogated in the copy number segment, gene copy number and methylation data. Furthermore, a combination of gene set enrichment analysis (GSEA) and pathway ontology gene ontology analysis (POEA) was used to investigate the enrichment of *NTRK1/2/3* signalling in the CRC cohorts. Moreover, the enrichment of kinase inhibitor response was interrogated in the CRC cohorts using the GSEA and the drug ontology enrichment analysis (DOEA).

### 4.2. Study Cohorts

This study included the Cancer Genome Atlas (TCGA, n = 537) and the Sidra-Leiden University Medical Center’s Atlas and Compass of Immune–Cancer–Microbiome (Sidra-LUMC, n = 348) CRC cohorts from the Genome Data Commons (GDC) and CBioPortal databases [20,21,22,23,24]. This study was retrospective in design, and the analysed data consisted of clinicopathological, RNASeq, masked copy number segment, gene copy number and methylation data. Three cases in the TCGA cohort had NTRK3 fusion, as shown in the structural variation data; these were excluded from analyses. Structural variation data was not available for the Sidra-LUMC cohort.

### 4.3. Data Retrieval and Processing

The clinical and genomic data of interest were extracted from the TCGA and the Sidra-LUMC dataset using the Windows-based Ubuntu 20.04 platform. Continuous variables, such as NTRK gene expression levels, were Winsorised at trimmed means of 15% (*NTRK 1* and *NTRK3*) and 30% (*NTRK2*) to mitigate the effect of outliers in both CRC datasets, before zero-to-one normalisation. Following zero-to-one normalisation, data from both CRC cohorts were combined into a single cohort. Cohort bias was tested using one-way ANOVA (continuous variables) and the chi-square test (categorical variables), while regression analysis was used to exclude cohort bias, as applicable. Gene set enrichment analysis (GSEA) was individually performed for the two CRC cohorts due to the unequal number of genes profiled for the individual datasets (TCGA = 60,483, and Sidra-LUMC = 18,355). Linux-based scripts were also used to prepare txt-formatted gene expression datasets, as per the GSEA requirements [25,26], while the phenotype and derivative gene set files were prepared in Excel spreadsheets and converted to cls and gct files, respectively.

### 4.4. Gene Set Enrichment Analysis with Gene Ontology Enrichment Analyses

The gene set enrichment analysis (GSEA) was performed using GSEA_4.3.3 software with the NTRK1/2/3 gene sets from the Harmonizome database (https://maayanlab.cloud/Harmonizome/ (accessed on 5 September 2025)) [27,28] to interrogate the enrichment of NTRK1/2/3 signalling in the CRC cohorts. The GSEA was validated with POEA on the Enrichr platform (https://maayanlab.cloud/Enrichr/ (accessed on 5 September 2025)) [29]. The GSEA and DOEA were also utilised to investigate the enrichment of kinase inhibitor responses in the CRC cohorts. Drug sets for multiple kinase inhibitors were retrieved from the Drug Signature database (https://dsigdb.tanlab.org/DSigDBv1.0/ (accessed on 5 September 2025)) [30] and incorporated into the analyses. The GSEA of multiple cohorts followed the requirements of MSigDB: the enriched genes from the analyses of the TCGA cohort were compiled into a gene set, which was then used to interrogate the Sidra-LUMC cohort.

### 4.5. Statistical Analyses and Data Visualization

SPSS version 29 was utilized to assess the clinicopathological, molecular, genomic and methylation correlates of *NTRK1/2/3* expression in CRC. Modified zero-to-one normalisation and the Templeton method were also performed using SPSS. Associations between categorical variables were investigated using the chi-square test, and correlations between continuous variables were analysed using bivariate correlation. A one-way ANOVA test was used to measure the mean differences of normalised continuous variables between discrete groups, while the median test for K samples was used for median differences when the continuous data could not be normalised. Multivariate analysis was investigated using regression analyses. A *p* value of <0.05 was taken as the threshold for a significant association or correlation. The Benjamini–Hochberg correction was applied for multiple testing using a false discovery rate (FDR) of 0.05. The GSEA was performed with default threshold nominal rate of 0.05 and an FDR of 0.25, as per MSigDB requirements. The permutation number was maintained as 1000, while permutation type was set as either “gene set” or “phenotype”, as appropriate. Figure 1 and Figure 4B were generated using tools from the online SR plots platform on https://www.bioinformatics.com.cn/en (accessed on 15 September 2025) [31]. Figure 2 and Figure 3 were generated from the SPSS version 29 statistical analyses, while Figure 5 was generated using the Flourish Studio platform (https://flourish.studio/product/data-visualization/, accessed on 15 September 2025) [32].

## 5. Conclusions

In conclusion, this study demonstrated that *NTRK1/2/3* overexpression outside the context of NTRK fusion exhibits associations with adverse clinicopathological and molecular features, as well as with enrichment of oncogenic signalling pathways, constituting evidence of their biological relevancies in the pathogenesis of CRC. Furthermore, this study demonstrated that copy number gains/amplifications and aberrant promoter methylation may be the main causes of *NTRK1/2/3* deregulation in CRC. In addition, *NTRK1/2/3* expression showed associations with responses to multiple kinase inhibitors, suggesting that *NTRK1/2/3* overexpression may be a biomarker of response to multiple kinase inhibitors, including NTRK inhibitors.

## Data Availability

All the genomic and clinicopathological data utilized for this study are freely available in the cBioPortal for Cancer Genomics website (https://www.cbioportal.org/, accessed on 2 August 2025) and the Genome Data Commons repository (https://portal.gdc.cancer.gov/, accessed on 2 August 2025).

## Supplementary Materials

The following supporting information can be downloaded at https://www.mdpi.com/article/10.3390/ph18101562/s1.

## Abbreviations

The following abbreviations are used in this manuscript:

TCGA	The Cancer Genome Atlas
Sidra-LUMC	Sidra-Leiden University Medical Center’s Atlas and Compass of Immune–Cancer–Microbiome
GDC	Genome Data Commons
GSEA	Gene set enrichment analysis
POEA	Pathway ontology enrichment analysis
DOEA	Drug ontology enrichment analysis
*NTRK1/2/3*	*NTRK1*, *NTRK2* and *NTRK3*
